# What Can N-glycomics and N-glycoproteomics of Cerebrospinal Fluid Tell Us about Alzheimer Disease?

**DOI:** 10.3390/biom11060858

**Published:** 2021-06-09

**Authors:** Stefan Gaunitz, Lars O. Tjernberg, Sophia Schedin-Weiss

**Affiliations:** 1 Department of Clinical Chemistry, Karolinska University Hospital, 14186 Stockholm, Sweden; rolf.gaunitz@sll.se; 2 Division of Neurogeriatrics, Department of Neurobiology, Care Sciences and Society, Karolinska Institutet, 17164 Solna, Sweden; lars.tjernberg@ki.se

**Keywords:** glycomics, glycoproteomics, N-glycan, Alzheimer disease, cerebrospinal fluid, mass spectrometry

## Abstract

Proteomics—large-scale studies of proteins—has over the last decade gained an enormous interest for studies aimed at revealing proteins and pathways involved in disease. To fully understand biological and pathological processes it is crucial to also include post-translational modifications in the “omics”. To this end, glycomics (identification and quantification of glycans enzymatically or chemically released from proteins) and glycoproteomics (identification and quantification of peptides/proteins with the glycans still attached) is gaining interest. The study of protein glycosylation requires a workflow that involves an array of sample preparation and analysis steps that needs to be carefully considered. Herein, we briefly touch upon important steps such as sample preparation and preconcentration, glycan release, glycan derivatization and quantification and advances in mass spectrometry that today are the work-horse for glycomics and glycoproteomics studies. Several proteins related to Alzheimer disease pathogenesis have altered protein glycosylation, and recent glycomics studies have shown differences in cerebrospinal fluid as well as in brain tissue in Alzheimer disease as compared to controls. In this review, we discuss these techniques and how they have been used to shed light on Alzheimer disease and to find glycan biomarkers in cerebrospinal fluid.

## 1. Introduction

The molecular profile of cerebrospinal fluid (CSF) can tell us a lot about the status of the brain and alterations associated with neurodegenerative diseases such as Alzheimer disease (AD). Importantly, such knowledge can be used for understanding disease mechanisms, and the differently expressed molecules can be used as biomarkers. Over the last decades, high-performance liquid chromatography (HPLC) coupled to tandem mass spectrometry (MS/MS) has proven to be an invaluable tool for large-scale studies of proteins—proteomics. The introduction of efficient labeling techniques and high-resolution MS/MS instruments has enabled identification and quantification of several thousands of proteins. Today, there are numerous such proteomics studies, but studies on post-translational modifications, including glycosylation, are scarce. Herein, we discuss technical aspects of glycomics and summarize the few reported glycomics studies of CSF in AD. Finally, we give an introduction to the emerging synthesis of proteomics and glycomics: glycoproteomics. Our aim is to emphasize that such studies are highly needed, since the available reports are few. The first in-depth site-specific N-glycoproteomics study of CSF samples from AD patients was only recently published [1], while there are enormous amounts of information that could be gained from such studies. 

## 2. Alzheimer Disease

Alzheimer disease (AD) is a devastating neurodegenerative disorder and the most common form of dementia. It is a global health problem and the current treatments are only symptomatic, and thus, there is an urgent need for a disease modifying drug [2].

AD pathology is characterized by two pathological hallmarks: extracellular amyloid plaques composed of the amyloid β-peptide (Aβ) [3] and dystrophic neurons filled with neurofibrillary tangles composed of tau protein [4], together with a progressive degeneration and loss of neurons and synapses. Aβ starts to accumulate in AD brain decades before symptoms appear, followed by tau hyperphosphorylation and tangle formation, and an inflammatory response [5]. Most of the therapeutic approaches tested have focused on these three pathologies. Although it is likely that Aβ oligomerization triggers the pathogenic process, resulting in tau phosphorylation and deposition, inflammation, synapse loss and neuronal death, the finer details of the mechanisms behind AD remain largely unknown. So far, Aβ has been the most popular target, and the clinical trials have aimed at either reducing its formation or increasing the clearance. Aβ generation can be reduced by inhibitors of either of the two enzymes that process the Aβ precursor protein (APP), a single-pass transmembrane protein involved in physiological processes such as neurite sprouting and cell–cell contacts [6]. The first cleavage step is mediated by β-secretase (beta-site APP cleaving enzyme 1, BACE1) resulting in the release of soluble APPβ and a membrane-bound 99-residues C-terminal fragment (C99) [7]. The second cleavage is mediated by the multi-transmembrane complex γ-secretase, which consists of the four proteins presenilin 1 or 2, nicastrin, anterior pharynx defective-1, and presenilin enhancer-2 [8]. Inhibitors of these enzymes have been tested in clinical trials and all have failed, in many cases due to the fact that both enzymes have a multitude of substrates of physiologic importance [7,8]. Thus, the inhibition of the secretases must be more specific for APP-processing. As described below, the two secretases as well as APP are glycosylated, and thus, glycans could be of importance for AD.

## 3. Importance of N-glycans in the Central Nervous System

**Figure 1:**
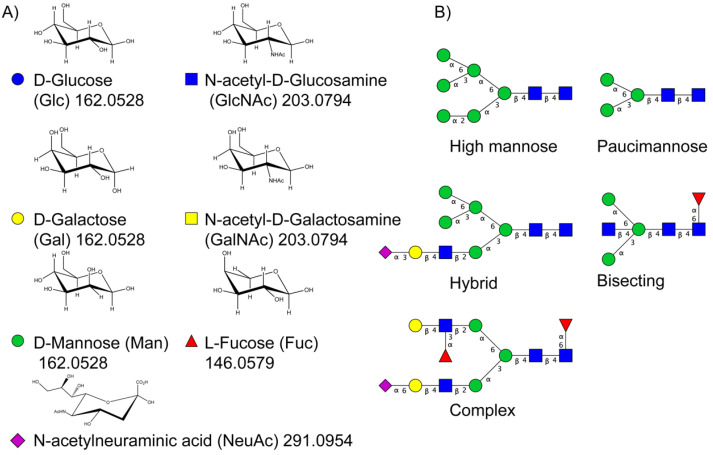
Schematic representations of monosaccharides and N-glycans drawn as chemical structures or cartoons. (**A**) Examples of common monosaccharides in human. The masses of the monosaccharide units, as part of a glycan, are shown. (**B**) Examples of N-glycan types. The chemical- and cartoon structures were drawn in ChemDraw and GlycoWorkBench [10], respectively and finalized in Adobe Illustrator.

Protein glycosylation is the most extensive and complex form of post-translational modification of proteins. Herein, we give a brief introduction, while for a deeper understanding, we refer to Essentials of Glycobiology [9]. In contrast to the protein sequence, which is coded by the genome, the N-glycan structures are not template derived and cannot be directly predicted from the genome and must be determined with analytical techniques. Glycans can theoretically be combined in an almost infinite number of ways, since the monosaccharide building blocks in vertebrates can exist in two configurations (D or L) and form bonds in different directions with each of its different hydroxyl groups. Typical vertebrate monosaccharide building blocks are shown in Figure 1A.

Glycan moieties of glycoproteins are crucial for several important functions including protein folding, quality control during protein translation, intracellular transport, cell–cell communication, cell–matrix interactions, cell adhesion, ligand–receptor interactions, host–pathogen interactions, and immunological responses.

Protein glycosylation can broadly be divided into two forms: O-glycosylation and N-glycosylation. For O-glycosylation, glycans are built onto a serine or threonine (or in rare cases tyrosine) of the protein backbone after translation. O-glycosylation can be further divided into different types, including mucin type of glycans, glycosaminoglycans and the addition of a single N-acetylglucosamine (GlcNAc) residue (so-called O-GlcNAcylation). In this review, we have focused on N-glycosylation and will not further discuss O-glycosylation. For N-glycosylation, a precursor is added en bloc to an asparagine (Asn) side chain in the consensus sequence Asn-X-Ser/Thr. This occurs in the endoplasmic reticulum (ER), usually on the growing polypeptide chain during protein translation. The glycan precursor is then trimmed and elongated during transport through the ER-Golgi system to a final mature form by various glycosyltransferases and glycosidases. These activities are easily disturbed. The glycans may therefore represent a phenotypic marker of a cell type or development stage and may differ in health and disease. All N-glycans share a common starting sequence composed of two GlcNAc residues elongated with three mannose residues that may be further elongated with various monosaccharides (Figure 1A). The N-glycan core may be decorated with additional mannose residues to form high-mannose glycans or additional fucose and sialic acid residues to form complex glycans or both mannose and GlcNAc, N-Acetylgalactosamin (GalNAc), sialic acid (N-acetylneuraminic acid, NeuAc) or fucose to form hybrid glycans (Figure 1B). The N-glycan can also be trimmed with specific glycosidases to form a truncated form, paucimannose. 

N-glycans are typically transported through the secretory pathway for secretion or for insertion into the plasma membrane, but they can also be found in intracellular organelles [9]. N-glycans play important roles in the central nervous system (CNS) and N-glycan epitopes (including human natural killer-1, poly-sialic acid, Lewis X, oligomannosidic glycans, fucose(α1-2)galactose, α2,3-sialic acid and bisecting GlcNAc) regulate for instance neuronal differentiation, neurogenesis and neuritogenesis, cell migration, synaptic activity, long term potentiation, long-term depression, memory formation, spatial learning and nerve regeneration (for reviews, see [11,12]). Interestingly, the functions described above are defective in neurodegenerative disorders, including AD. Thus, it is highly relevant to further investigate the link between protein glycosylation and AD. Here, we summarize what is known about N-glycomics and N-glycoproteomics studies of CSF in AD and what we can learn from those findings.

## 4. Biomarkers for Alzheimer Disease

Accumulating evidence suggest that the disease begins ~20 years before the appearance of symptoms [13], and it is possible that some of the drugs previously tested in clinical trials would be effective if given at an earlier stage of the disease. Thus, it is important to find ways to diagnose the disease presymptomatically. AD is a heterogeneous disease with variants that may be differently affected by pharmaceutical treatments, and there are overlapping symptoms between AD and other neurodegenerative disorders. Hence, for enhanced success in clinical trials and AD treatments, it is necessary to: (1) diagnose AD cases earlier and more accurately, (2) identify subpopulations of AD, (3) enrich and stratify populations for clinical trials and (4) reliably monitor disease progression and response to treatment.

Biomarkers currently used for AD include concentrations of proteins, peptides or other molecules in CSF or blood [5,14]. Brain imaging techniques such as magnetic resonance imaging (MRI) and positron emission tomography (PET) are also used but are invasive, expensive and can only be performed at specialized hospitals. CSF is in direct contact with the brain, and the levels of molecules in CSF partly reflect the levels in the brain. The CSF biomarkers total tau (T-tau), phospho-tau (P-tau) and Aβ42 can distinguish AD patients from control individuals and are used in clinical trials and AD diagnostic procedures [5,14]. Blood tests are more easily taken, but the levels of molecules in blood are less representative than CSF levels for reflecting alterations in brain. Moreover, the relatively low levels of CNS-derived proteins in blood, as well as extra-CNS production of the analytes, complicate the interpretation. Recent articles have described the use of P-tau in blood as a potential biomarker [15,16]. Blood-based biomarkers may thus have a high potential to be used for initial screening purposes in large populations. Still, considerable efforts are needed to find novel biomarkers that can identify different subgroups and stages of AD, enable earlier diagnosis and be used to improve clinical trials and treatments for AD, and most likely CSF biomarkers have higher potential in this respect. Most fluid biomarkers are so far strictly based on proteins or fragments thereof, and post-translational modifications other than phosphorylation rarely considered. However, the emerging fields of glycomics and glycoproteomics may offer novel biomarkers for early diagnosis and subgrouping and, possibly, insights to disease mechanisms and treatment strategies.

## 5. Alterations in Glycosylation of Pathogenic Proteins in Alzheimer Disease

Several recent reviews have described alterations in glycosylation of individual proteins or classes of proteins in AD (see for instance [17,18,19,20]). Here, we briefly summarize some of these findings to emphasize the importance of including glycans upon evaluation of the structures/functions of glycoproteins involved in AD pathogenesis.

### 5.1. Proteins of the Amyloidogenic Pathway

Most components of the amyloidogenic APP processing pathway are glycoproteins, and their glycosylation patterns have been found to be altered in AD. APP is a type 1 transmembrane protein with two N-glycosylation sites that are located at the extracellular/lumenal domain of the protein [21,22,23]. The N-glycans are important for proper intracellular sorting of APP, including axonal transport, as well as processing and secretion of APP fragments that are released upon processing by secretases. APP also has several O-glycosylation sites that have been reported to affect APP processing and the secretion of Aβ [24,25,26]. The fact that APP in CSF is both N- and O-glycosylated supports the notion that both these pathways are active upon transport of APP through the secretory pathway. Still, the roles of the individual glycosylation sites remain to be determined. Interestingly, the glycosylation pattern of secretory APPα has been suggested to be altered in AD brain [27].

BACE1 is a type 1 transmembrane protein with four N-glycosylation sites but devoid of O-glycosylation sites. The N-glycans are required for the folding and maturation of BACE1. The presence of bisecting GlcNAc-bearing N-glycans on BACE1 has been reported in human and mouse brain [28]. The expression of such glycans was increased in BACE1 from AD patients compared to controls. Studies in mice showed that presence of bisecting GlcNAc on BACE1 targets the enzyme to early endosomes, where it can cleave APP. In the absence of bisecting GlcNAc, the enzyme is targeted to late endosomes/lysosomes, where it is degraded. In line with these findings, bisected glycans on BACE1 enhance the formation of Aβ. In addition to APP, BACE1 can process a range of other substrates, including sialyltransferases, such as ST6GAL1 and can thus directly influence glycosylation via its secretase activities [29,30]. The effect of bisecting GlcNAc on APP processing but not on processing of two other substrates, neural cell adhesion molecule L1-like protein (CHL1) and contactin 2, was affected by bisecting GlcNAc, suggesting that BACE1 cleaves those substrates and APP in different compartments [18,28].

**Figure 2:**
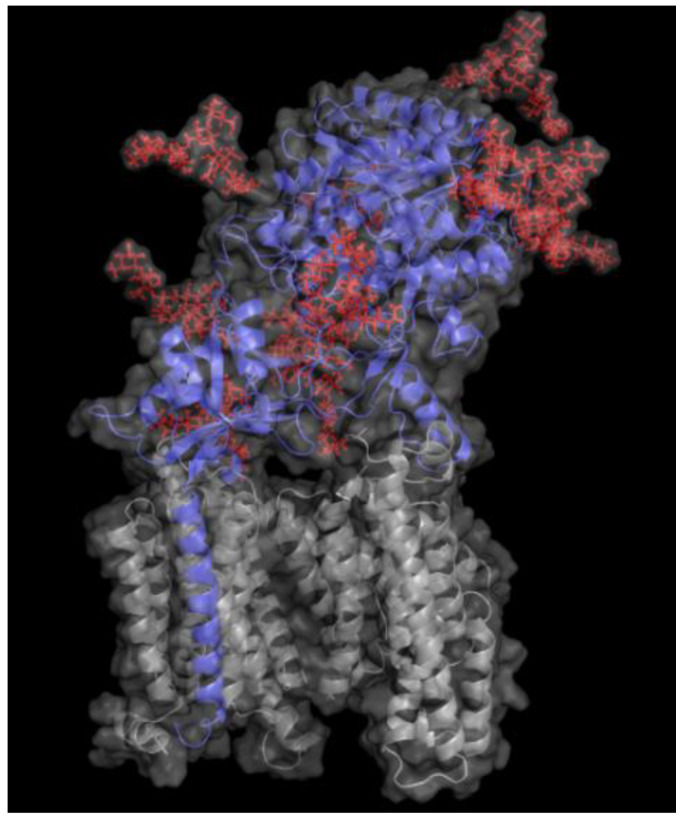
γ-Secretase structure with 11 N-glycans modeled on nicastrin. Ribbon representation of human γ-secretase is shown with the nicastrin unit marked in blue and presenilin, anterior pharynx defective-1 and presenilin enhancer-2 marked in grey. Eleven of the 16 potential N-glycosylation sites are modelled in red. The 11 glycans were chosen based on the fact that the cryo-EM structure showed parts of these glycans. Still, it is likely that all N-glycosylation sites can be occupied. The modelled N-glycans are simple biantennary hybrid and high-mannose structures. It is likely that more complex glycans are additionally present on nicastrin in human brain. The structure was drawn based on the Cryo-EM structure of γ-secretase using pdb coordinates 5A63 [42]. The glycans were modeled with CHARMM-GUI [43] using glycan reader and modeler [44,45,46]. The structure was visualized using The PyMol Molecular Graphics System, Version 2.5.0, Schrodinger, LLC.

Among the four protein components of γ-secretase, only nicastrin is glycosylated, containing as much as 16 potential N-glycosylation sites. These N-glycosylation sites are present on the large extracellular/lumenal domain of nicastrin [31,32,33]. Much remains to be determined regarding the physiological roles of these glycans. However, a recent study showed that the N-glycans of nicastrin influence the catalytic activity and substrate preference of γ-secretase [34]. Moreover, the impact of so many N-glycans on the protein surface (Figure 2) is likely to affect recognition and interaction with ligands, substrates and other molecules. While presenilin is not glycosylated, lack of presenilin was found to result in defective glycosylation of several proteins, including nicastrin, NCAM and tyrosine-related kinase B [33,35,36,37,38], suggesting that PS1 can regulate protein glycosylation either directly or indirectly. Notably, another protein that has been reported to regulate complex glycosylation, cell surface expression and secretion of APP is the type I transmembrane Golgi-resident protein TMEM59 [39]. Conversely, the final product of the amyloidogenic machinery, Aβ, affects protein glycosylation, since Aβ exposure, for instance, leads to upregulation of GnT-III in neuroblastoma cells [40] and affects glycosylation of reelin [41].

### 5.2. Other Proteins of Interest

Interestingly, tau, which has three potential N-glycosylation sites, has been reported to be glycosylated in AD brain but not control brain [47,48,49]. Since tau is a cytosolic protein, it is not expected to be N-glycosylated. However, tau has also been found in the plasma membrane and in CSF, where its level is increased in AD [50,51].

The surface receptor triggering receptor expressed on myeloid cells 2 (TREM29, a protein expressed by microglia), is involved in the regulation of inflammation and phagocytosis. Genetic variants of TREM2 are risk factors for AD, and some of these variants have altered glycosylation [52].

Acetylcholinesterase, an enzyme that degrades the neurotransmitter acetylcholine and terminates synaptic transmission at cholinergic synapses, has three potential N-glycosylation sites. Alterations in glycosylation of acetylcholinesterase has been reported both in brain and in CSF in AD [53,54]. Similarly, the related compound butyrylcholinesterase has altered glycosylation in AD [55,56].

Several additional glycoproteins have been reported to have altered N-glycosylation in CSF in AD, including transferrin [57] and reelin [58], and the number of such proteins keeps increasing.

One possible explanation of these alterations could be a dysregulation of glycosyltransferases in AD. This notion is strengthened by the finding that a dysregulation of several protein glycosylation pathways in AD was described in a study comparing mRNA expression of glycosyltransferases in control and AD brains [59]. This study showed that some glycosyltransferases are upregulated, while others are downregulated in AD brain. Other examples of altered expression if glycosyltransferases in AD include downregulation of sialyltransferases in neurons [60] and increased activity of MGATIII, the enzyme responsible for synthesis of bisecting GlcNAc, in AD brain [40].

## 6. Glycomics Studies of Cerebrospinal Fluid in Alzheimer Disease

Unbiased searches for new glycoprotein biomarkers involve screening for changes in glycosylation of a glycoprotein in a patient sample such as blood, urine or CSF. Identifying the peptide, glycosylation site and glycan composition/sequence of all glycopeptides in a complex sample is technically challenging. Therefore, glycoprofiling, the MS profiling of enzymatically removed N-glycans, has been the viable option in many biomarker studies of various diseases. However, with this strategy the link to the protein is lost, and the function of the glycans that are found to correlate with a disease state cannot easily be understood, since the link to any specific protein is lost. Most glycocentric biomarker studies on AD have been focusing on glycomics, but a recent large scale glycoproteomics study demonstrates that the technical challenges are now rapidly being overcome [1]. Below, we summarize the glycomics and glycoproteomics studies hitherto performed on CSF from AD patients and controls in Section 6.2 and Section 7.5, respectively.

### 6.1. Workflow of Glycomics

No single analytical method can be used to confidently assign all structural information of N-glycans, since they are so information-rich molecules due their branched structure, isobaric monosaccharide components and the anomericity and linkage variability of the glycosidic bonds between the monosaccharide components in the oligosaccharide chain. The theoretical number of unique oligosaccharide structures that can be produced in humans has been calculated to be almost astronomical [61]. Thankfully, the number of detected structures are much smaller, and estimates have been in the range of 10,000 glycans [61].

The facile release of N-glycans from proteins with the highly specific enzyme PNGase F, the conserved N-glycan core structure and the N-glycosylation site amino acid consensus sequence have greatly facilitated the study of N-glycans [62].

Native N-glycans are hydrophilic and can be separated with hydrophilic interaction liquid chromatography (HILIC) (normal phase) or reversed-phase columns such as porous graphitized carbon (PGC). PGC chromatography of N-glycans can yield isomeric separation based on size, charge and N-glycan structure. As the N-glycan structure becomes larger and carries more charge (more sialic acid residues), the separation becomes less efficient, and a longer column or derivatization of the sialic acid becomes necessary [63].

Glycans are often derivatized prior to mass spectrometric analysis. Permethylation, the chemical addition of methyl groups to every hydroxyl group of the glycan, is one of the most common derivatization strategies. The workflow needs careful calibration to ensure near complete conversion of all hydroxyl groups, but when done correctly it confers the advantage of increased stability and ion intensity in MS analysis [64,65]. In addition, permethylation mitigates the problem that different N-glycans have different ionization efficiency when analyzed in native form. This makes relative quantification in MS glycan profiling more accurate. Permethylation also simplifies generation of MS2 spectra containing fragment ions that can give cues to the linkage. Permethylated glycans may be separated on reversed-phase C18 or porous graphitized carbon (PGC) columns coupled to MS. Isomeric separation of permathylated N-glycans has been observed on PGC [63]. Reductive amination of glycans with the labels 2-anthranilic acid (2-AA) or 2-aminobenzamide (2-AB) facilitates chromatographic separation on C18 columns and enhances the MS signal. HILIC or C18 columns are suitable for AA- or AB-labeled glycans. The quantitative performance of four high-throughput glycomics methods was compared; LC with fluorescence detection, capillary electrophoresis with fluorescence detection, matrix-assisted laser desorption/ionization (MALDI)-time-of-flight (TOF) and LC-MS of glycopeptides [66]. All methods were deemed suitable for quantitative studies, but loss of sialic acid was observed in the MALDI MS analysis of native N-glycans. Stabilization of sialic acid with, for example, permethylation is thus necessary to avoid this artefact. Relative quantification comparing the glycan MS profile of different samples requires normalization, and the choice of method influences the results greatly [67].

### 6.2. Glycomics Studies of Cerebrospinal Fluid in Alzheimer Disease

There are a few recent glycomics studies in which mass spectrometry was used to study alterations in the N-glycome in CSF in AD compared to controls [68,69,70,71]. These studies have in common that glycans were released enzymatically by PNGase F treatment prior to derivatization and mass spectrometric analysis. One study used MALDI MS techniques to analyze permethylated N-glycans in CSF from 24 AD patients, 11 cases with mild cognitive impairment (MCI) and 21 healthy controls [68]. Overall, they found a decrease in the sialyation degree and an increase in species containing bisecting GlcNAc in 40–50% of the MCI and AD cases. Increased levels of bisected N-glycans and overall decrease in sialylated glycans were also observed in another recent CSF glycomics study, in which pooled samples from three groups (31 control subjects, 27 MCI cases and 25 AD cases) were analyzed by MALDI-TOF MS of permethylated glycans [71]. In this study, LC-MS/MS ESI was also used to analyze N-glycans derivatized with 2-anthranilic acid (2-AA) of individual CSF samples from five AD patients and five age- and sex-matched controls [71]. An increase in bisected N-glycans was confirmed by both approaches. Regarding the levels of sialylated N-glycans, a downregulation was observed in a subpopulation of the AD cases in the LC-MS/MS study [71].

Another study used LC-MS and permethylated glycans to analyze CSF samples from four female controls, four female AD, four male controls and four male AD patients [70]. Results from control female and control male individuals could be distinctly separated by principal component analysis (PCA) plots [70]. PCA plots could also separate between AD and controls when the genders were analyzed separately but not combined [70]. This study revealed 15 glycans with altered levels in female AD patients compared to controls and 10 glycans with altered levels in male AD patients compared to controls. Interestingly, there was only a partial overlap between the N-glycans with altered levels in AD when the male and female groups were compared. For male individuals, decreased levels of a number of hybrid glycans was observed in AD [70].

One study explored a glycoblotting technique, employing BlotGlyco^®^ beads to capture glycans, followed by derivatization using an O-benzylhydroxylamine-based labeling method and MALDI-TOF MS to analyze glycans from CSF of controls and AD patients with 2–3 male or female individuals in each group [69]. This study differed from the studies described above by reporting an overall increase in N-glycans in CSF from AD males and females compared with the corresponding control groups. The most prominently increased glycoforms in AD were those bearing bisecting-GlcNAc, proximal fucose and tri- and tetra-antennary N-glycans.

For certain glycans, the direction of alterations is inconsistent between the studies. This could reflect methodological differences as well as individual differences due to the heterogeneity of AD. The studies from Palmigiano [68] and Schedin-Weiss [71] included different stages of cognitive impairment and could thus show that glycan changes occur early during disease progression. Palmigiano et al. [68] reported that patients with MCI, which in many cases represents a pre-stage of AD, could be divided into two groups—those with an atypical glycosylation and those with a normal glycosylation. All cases in the group with atypical glycosylation converted to AD, suggesting that the altered glycan profile represents a risk factor for individuals who will develop AD. In line with the Palmigiano study, Schedin-Weiss et al. [71] showed that the alteration in glycomics pattern in CSF at the MCI stage was at least as prominent as for AD. The studies from Gizaw [69] and Cho [70] showed gender-specific glycan profiles, underscoring the importance of analyzing the sexes individually. It would be interesting to further explore how glycan levels correlate with clinical parameters and other biomarkers in larger cohorts in future studies. A summary of alterations of glycan levels reported from glycomics studies of CSF in AD described above is shown in Table 1.

Comparing the results from the different glycomics studies of CSF in AD, it is clear that increased levels of bisected N-glycans is the most consistent finding. To further explore the expression of bisected N-glycans in CSF in AD, and simplify analyses of large cohorts, a lectin-linked enzyme assay (ELLA) in multiwell-plate format was developed [71]. The validity of this assay was verified by analyzing CSF from well-defined AD cases and age- and sex-matched controls, confirming that AD cases have significantly increased levels of this epitope. Moreover, a cohort of 242 patients with different stages of cognitive impairment, (i.e., subjective cognitive impairment (SCI), MCI and AD), was analyzed with ELLA, showing increased levels of bisected N-glycans in AD compared to SCI [59]. Intriguingly, the levels of bisected N-glycans measured by ELLA correlated with CSF levels of total tau and P-tau, and this correlation was particularly pronounced in the SCI group. Since SCI cases in many cases develop AD, these findings indicate that bisected N-glycans may represent a very early biomarker for AD and should be further explored in future longitudinal studies.

The atypical glycomics pattern in brain differs from that in CSF in AD [69,72]. Considering the fact that glycans function as address tags, these differences could be explained by a potentially altered extent of secretion of glycoproteins in AD brain. The altered glycosylation reported in AD brain may thus affect vesicular transport and glycoprotein secretion. Alternatively, the differences in the glycan profile alterations in brain and CSF could also reflect that CSF contains molecules derived from dead cells. It is interesting to note that a large-scale study determining alterations in glycosylation site occupancy in AD brain revealed several dysregulated N-glycosylation-affected processes and pathways, including neuroinflammation, synaptic dysfunction, alterations in cell adhesion, lysosomal dysfunction, dysregulation of endocytic trafficking, dysfunction of ER and others [73]. To obtain a clearer picture about the mechanisms behind the altered glycosylation profile in AD, we need to analyze the glycoproteome to determine which glycans are attached to which glycosites of which individual glycoproteins.

## 7. Glycoproteomics

LC-MS based glycopeptide analysis was previously limited in complexity and scope, but recent studies have successfully reported thousands of unique glycopeptides derived from hundreds of glycoproteins (reviewed in [74]). A summary of N-glycomics and N-glycoproteomics studies in human CSF are presented in Table S1. The most comprehensive glycoprotemics study conducted so far on CSF from AD patients was recently published by Chen et al. and can serve as an example of a successful analytical strategy when we summarize workflow and promising glycoproteomics techniques below [1].

### 7.1. Generation of Glycopeptides

The workflow of generation of glycopeptides is usually not different from a standard proteomics workflow. In addition to tryptic digestion in solution, filter-aided sample preparation (FASP) and variations thereof have successfully been integrated in several glycoproteomics workflows [1,72,73]. In brief, the use of molecular weight cutoff (MWCO) spin filters allows the sequential denaturation, buffer exchange and tryptic digestion of proteins in a small reaction chamber that allows separation of proteins and salts. After denaturation, reduction and alkylation, trypsin is added. The resulting peptides and glycopeptides are collected in the effluent after spinning. In a typical sample digested with trypsin, only 1–5% of the peptides are glycopeptides. It is therefore highly advantageous to enrich the glycopeptides prior to analysis. In addition, even though the protein levels in certain body fluids, including CSF and urine, are significantly lower than in blood, the removal of abundant proteins, for instance albumin, which is not N-glycosylated, with immuno-depletion is often necessary and has been suggested by several groups [75,76].

### 7.2. Enrichment of Glycopeptides

For a complex sample, the enrichment of glycopeptides is challenging. Firstly, a rather large starting material is currently needed, since most of the peptides are not glycosylated and thus, up to 99% of the starting material may be lost in this step. The enrichment may be performed in a single step or in several sequential steps utilizing different techniques. A comprehensive review was recently published, listing the different kinds of glycopeptide enrichment strategies that are currently available and their applicability [77]. It is beyond the scope of this review to go through all the methods in detail. However, when choosing the appropriate enrichment strategy, several questions must be considered. Should the enrichment be broad or targeted? What is the enrichment efficiency (selectivity vs. nonglycosylated peptides)? What is the ease of use and the complexity of the sample? The hydrazide, boronic acid and other chemistries used to conjugate glycans and glycopeptides to a solid resin should here be highlighted since they allow more stringent removal of nonglycosylated peptides and are both broad in their glycan specificities and highly selective for glycosylated peptides. Boronic acid enrichment was recently successfully used by Chen et al. in a glycoproteomic study of CSF [1]. To be able to perform a deep glycoproteomics study of CSF, Chen et al. resorted to pooling individual CSF samples since a large fraction of the peptides were removed under the stringent enrichment conditions employed. Although a popular choice, HILIC chromatography does not discriminate with high specificity between glycopeptides and hydrophilic peptides and therefore often results in a higher degree of nonglycosylated peptides left in the enriched sample [77]. Lectins, which to a varying degree recognize specific glycan epitopes, are also often used to enrich glycopeptides. A single highly specific lectin conjugated to agarose beads can be used for a targeted approach [78]. Alternatively, a battery of lectins covering most common glycan epitopes can be used as an untargeted approach [79]. Monoclonal antibodies can also be used for highly specific and selective targeted enrichment if these are available. Due to the complexity of the glycopeptide sample, fractionation is often needed to facilitate the downstream LC-MS analysis. High pH fractionation with a standard C18 column and/or isoelectric focusing fractionization and SCX or SAX are common protocols [1,80] and was indeed employed by Chen et al. prior to analytical separation and MS detection [1]. Injection of one fraction at a time allows for better chromatographic separation of the peptides and longer time to do MSn fragmentation resulting in deeper coverage.

### 7.3. LC-MS of Glycopeptides

From a chromatographic point of view, the glycan moiety makes the peptides more hydrophilic, which reduces their binding to a C18 reversed-phase column that is commonly used for peptide separations prior to MS detection in proteomic analysis. Nevertheless, most peptides can be separated with this column, although the use of HILIC and graphitized carbon columns have been reported. Standard mobile phases (water and acetonitrile) and gradients may be used, and the major difference between proteomics and glycoproteomics are the fragmentation settings and strategies (Table 2).

Collision-induced dissociation (CID) (iontrap) and higher-energy C trap dissociation (HCD, orbitrap) are suitable for glycomics where CID MSn and HCD MS2 fragments of glycans yield composition, sequence and structural information [81]. However, CID preferentially cleaves glycopeptides at the glycosidic bonds, which facilitates glycan characterization but leaves the peptide intact yielding only a blind mass. HCD and beam type CID (Q-TOF) MS/MS have in previous studies demonstrated peptide identification and partial glycan information but less information about glycosylation site. In recent studies where HCD or beam type CID were used with a ramp of MS fragmentation energies (energy-resolved HCD, stepped collision energy, sceHCD), information-rich fragments from both the glycan and peptide moiety resulted in peptide identification and glycan sequence as well [82,83,84,85,86]. Stepped collision energy HCD has in practice been as effective in identifying glycopeptides as more advanced fragmentation strategies combining several complementary techniques. Traditionally, MS instruments equipped with complementary fragmentation techniques have been used in order to generate fragments of both the peptide and glycan moiety of glycopeptides including information about the glycosite [96]. Glycosite information is crucial since it gives information about the various glycan structures at each site (microheterogeneity) and the occupancy degree of each site (macroheterogeneity). The function of glycosylation is partly known for some well-studied proteins but largely unknown for most proteins. Large-scale glycoproteomics experiments may reveal general patterns and give functional clues of glycosylation, based on glycan structural details and glycosite information. Electron transfer dissociation (ETD) and electron capture dissociation (ECD) generate peptide backbone fragments and peptide sequence information [89,90,91,92,93] but leave the glycan intact with a blind mass. Coupled with CID or HCD, a more complete annotation of the glycopeptide is possible [89,90,91,92,93,94,95]. The latest generation of tribrid systems such as Fusion Lumos from Thermo provides a wealth of fragmentation options such as CID, HCD, ETD EThCD and ETcID. The two latter techniques result in an MS2 spectra with daughter ions collected from both an HCD or CID and ETD fragmentation event. The suitability of these techniques has recently been systematically compared when analyzing N-glycopeptides and O-glycopeptides [88,96]. A common strategy is to first perform HCD survey scans and look for diagnostic oxonium ions in the MS2 spectra to identify glycopeptides that will be selected for EThCD fragmentation. This is a feasible strategy since the glycopeptide enrichment is often not complete, and many nonglycosylated peptides are still in the sample. Chen et al. analyzed glycopeptides separated on a capillary C18 column coupled to a tribrid Fusion Lumos instrument with a nanosource interface [1]. EThCD was conducted on all major peptides to generate high-quality MS2 fragments of both the glycan and peptide moiety of the glycopeptides. Glycoproteomic LC-MS analysis using sceHCD of glycopeptides from samples with limited complexity such as IgG, Fibrinogen, lactotransferrin and ribonuclease B can yield more in-depth knowledge of both peptide sequence and the glycan sequence including specific epitopes such as antennary GlcNAc, bisecting GlcNAc, hybrid- and high-mannose [97].

### 7.4. Data Analysis of Glycoproteomics Results

The large-scale glycoproteomic data analysis has continuously evolved, and reports of the complete sub glycoproteome of defined cells, body fluids or tissues are now becoming more common in both healthy and disease state, as reviewed by Thaysen et al. [62]. The data analysis has been a bottle neck and has a higher error rate than standard proteomics since both the glycan composition, site and peptide need to be correctly assigned. This is now being addressed by a number of software and databases. These tools were recently covered in comprehensive reviews [74,98,99,100]. A selection of software is listed in Table 3.

A testament to the rapid development of the glycoprotemics field is the incredible increase in scope of the Human Proteome Organization (HUPO) glycomics initiatives ranging from 2004 until today. HUPO has conducted several glycoproteomics community benchmarking studies and aims to identify the most appropriate analytical approaches. Due to technical restraints, the first studies only compared the released glycan moiety from pure glycoproteins. The latest HUPO human glycoproteomics initiative community study has a vast scope. High-quality LC-MS/MS- based glycoproteomics datasets of N- and O-glycopeptides from serum proteins were sent to 22 teams. From two datasets containing 16,445 and 18,444 MS/MS spectra (HCD, ETciD, EThCD and CID), glycopeptide identifications were reported for about a fifth of the spectra (3402 and 4982 from each dataset). A consensus list of 163 N-glycopeptides and 23 O-glycopeptides were reported by most teams.

Eleven search engines were employed for glycopeptide identification, of which 9 were glycopeptide-centric. Byonic was the most popular (n = 10) search engine.

Several high-performing informatics tools were identified, although the discrepancy was large between different informatics solutions. The top three search engines for N-glycopeptide identification were Byonic, Protein prospector and GlycoPAT, which received similar scores (Table 3). In addition, Byonic and Protein prospector were also top-ranked for O-glycopeptide identification. Two significant search engines were not included in the study due to data incompatibility (pGlyco [86] and recently released MSFragger-glyco [102], Table 3). Most search engines search for potential glycans as variable modifications on all potential glycosylation sites. This may result in a very large search space and consequently long processing times. An open search involves determining the peptide moiety by matching fragment ions without knowledge of the precursor glycopeptide. The difference between the identified peptide mass and the observed precursor glycopeptide mass (delta mass) is the mass of the glycan. Some search engines search for mass offset matching glycan masses to score glycopeptides prior to a proteomics search (pGlyco). MSFragger-glyco combines open search with ion-indexed search algorithms that significantly reduce the processing time [102,107].

Both commercial and academic glycoproteomics high-quality software are thus now available for the community and can easily be integrated into the workflow of different platforms [108]. Prevailing challenges are a high false discovery rate of glycopeptides, the lack of standardization of the data collection and reporting and incomplete integration with other omics disciplines [62]. Chen et al. analyzed the glycoproteomic profile of pooled CSF samples from AD patients and controls using the commercial software Byonic and identified a massive 28,932 glycopeptides and 5113 glycosites on 285 glycoproteins [1]. The built-in database containing 182 N-glycan compositions was used to identify glycans based on composition. In total, 72 N-glycan compositions were identified on the glycopeptides in CSF. Since the database is mostly based on serum glycoproteins, a larger glycan coverage could have been possible by using a CSF specific glycan database based on their own glycomics study or based on previous publications.

### 7.5. LC-MS Glycoproteomics Studies of Cerebrospinal Fluid

Due to technical restraints, few glycoproteomics studies on native glycopeptides have been conducted so far on CSF and even less on CSF from AD patients. In this section, we briefly describe the glycoproteomic LC-MS studies conducted so far. Further details of experimental details are found in Table S1. Some studies have solved the technical problem of analyzing glycopeptides by analyzing the glycan and peptide moiety separately in the same study or only focusing on the peptide moiety. In the largest study conducted so far on deglycosylated peptides, Guldbrandsen et al. studied CSF from healthy donors after PNGase F removal of the N-glycans from the glycopeptides. The authors identified 1121 formerly glycosylated peptides (28,811 peptides in total) and 846 glycosites on 520 glycoproteins (3081 proteins in total) [109]. In corresponding plasma samples, 1050 proteins were identified, of which 877 proteins overlapped with CSF, while 2204 were exclusive for CSF and 173 were unique for plasma. This study may serve as a foundation for future studies, but the lack of glycan information precludes glycoform information and microheterogeneity for each site. Pan et al. analyzed CSF from AD in a pioneering study on the glycoproteome of CSF [110]. Using a complementary proteomics approach, deglycosylated and glycosylated peptides were prepared in parallel from a CSF pool of healthy volunteers. The two datasets were prepared with either hydrazide resin or lectin affinity enrichment of proteins prior to tryptic digestion and/or PNGase F treatment. In total, 216 glycoproteins were identified, but the glycosylation sites and the removed N-glycans were not identified. Nilsson et al. analyzed hydrazide enriched N- and O-glycopeptides derived from pooled CSF samples [111]. Due to the acid release from hydrazide beads, sialic acid was lost. In total, 36 N-linked glycosites were identified on 23 glycoproteins and similar amounts of O-glycopeptides. Five unique N-glycans were identified. Although modest in scope, this study successfully identified both glycosites and glycan moieties of native glycopeptides in a single LC-MS strategy using HCD fragmentation technique of an Orbitrap MS system, further discussed below. Goyallon et al. used three approaches to analyze the glycan profile and glycoproteome of CSF [112]. Released and permethylated N-glycans were analyzed with MALDI TOF MS. HILIC-enriched glycopeptides were analyzed in native or deglycosylated form after PNGase F treatment. A total of 124 glycopeptides on 55 glycoproteins were identified on 36 glycosites. Forty-nine PNGase F released and permethylated N-glycans were identified with MALDI TOF MS. By merging the datasets of glycosylated and deglycosylated peptides, 144 glycosites on 81 glycoproteins were identified. Using CID, the authors assigned glycan moiety structures with high fidelity and discussed the glycosylation of the major proteins, but this LC-MS strategy could not analyze native glycopeptides in a single experiment since CID gives scarce peptide backbone fragments resulting in low glycosite coverage (further LC-MS strategies are described in Section 7.3).

The most recent and most comprehensive glycoproteomics site-specific study conducted so far on CSF from AD patients was recently published by Chen et al. [1]. Glycopeptides were enriched with HILIC or boronic acid solid phase and prefractioned with high-pH fractionation prior to LC-MS/MS utilizing the advanced complementary MS fragmentation technique EThCD. This technique can yield information-rich MS/MS spectra with both glycan and peptide information (see Section 7.3). Pooled CSF from controls and AD patients was analyzed in parallel. In control CSF, as many as 2893 glycopeptides from 511 glycoproteins on 285 glycosites were identified analyzing native glycopeptides. In comparison, 2847 glycopeptides from 272 glycoproteins were identified in the AD CSF. In AD CSF, unique identifications included 114 N-glycosites and 46 N-glycoproteins, while unique identifications in CSF controls included 138 N-glycosites and 59 N-glycoproteins. This subset of unique proteoforms in AD CSF could harbor potential novel biomarkers of AD. From this list of altered glycoproteins, the authors discuss in detail the glycosylation and potential link to AD pathogenesis based on literature. Alpha-1-antichymotrypsin (ACT), ephrin-A3 and carnosinase CN1 displayed large differences in site occupancy and/or glycoforms and seem to be linked to the pathogenesis of AD, although it should be noted that the use of pooled samples prevents observations of disease heterogeneity and individual differences.

A recent publication by Zhang et al. may point to the future of functional understanding of protein glycosylation [73]. Zhang et al. performed a quantitative glycoproteome study with the focus on N-glycosite occupancy in AD brain and analyzed the data extensively and identified disease signatures and glycoprotein networks. This is a major step toward a functional understanding of the N-glycosylation in brain function in health and disease.

## 8. Conclusions and Outlook

Despite the recent advances in glycomics and glycoproteomics described above, we are just at the beginning of the glycomics and glycoproteomics era. We envision that technical developments, improved search algorithms and compilation of extensive databases will move the field forward.

Among the few N-glycomics reports available on CSF from AD patients, it has been concluded that some glycans have altered levels in AD. These findings could already today be implemented as biomarkers in a clinical setting. Still, there is much missing information until we have the complete picture about the overall glycomics changes in CSF in AD. Two studies reported on differences between male and female patients, and two other studies reported that the alterations occur early during disease progression. However, future studies involving larger cohorts and correlations of different glycans with sex, disease stage, clinical parameters and various other AD biomarkers are necessary. Such studies will be useful for further biomarker development. AD is a multifactorial disease, and thus, glycomics could potentially be helpful in subgrouping the disease by analyzing individual glycan epitopes that correlate with different stages or aspects of the disease. This would improve the selection for clinical trials and allow for personalized medicine.

Fully understanding the glycomics changes in AD cold also be used for novel treatment strategies of the disease. For instance, since altered expression of several glycosyltransferases have been reported in AD, targeting these enzymes with glycan-mimicking compounds could be a potential treatment strategy. However, it is highly possible that alterations in glycan expression differs for different proteins and, while a certain glycan may be pathogenic for one protein, it may be physiologically important for another protein. Therefore, we need to be able to (1) determine the glycan occupancy and glycan structure of each potential glycosylation site of each protein and (2) understand the functions of each glycan of all proteins.

By combining proteomics and glycomics into glycoproteomics, the complexity—but also the possibilities—increase enormously. Thus, the alterations in protein glycosylation could truly be determined and give novel clues to pathogenic mechanisms and suggest novel strategies for treatment. It is interesting to note that the glycomics changes in AD brain differ from those in CSF. One potential explanation to this observation could be that the altered N-glycan profile in CSF is caused by altered secretion of certain glycoproteins in AD compared to control, but many other explanations are also plausible. Thus, glycoproteomics analysis of CSF as well as different regions and cell types in brain will contribute to understanding the link between protein glycosylation and AD.

The information gained could also be used for diagnostic purposes. Once the glycosylation sites dysregulated in disease have been determined, simple assays could be developed. For instance, in a multiwell-plate format, the capturing could be directed to the protein, and a lectin directed to the glycan could be used for detection. Such an approach could be used also for other neurodegenerative diseases, for instance Parkinson’s disease.

In clinical practice, blood samples are preferable to CSF samples. To this end, it is of interest to note that certain N-glycan structures are brain specific. Thus, by analyzing brain-specific glycans or glycopeptides in blood, the results will not be confounded by peripherally expressed proteins. Since development of AD and many other neurodegenerative diseases start decades before the appearance of symptoms, a simple screen for pre-symptomatic diagnosis may be required for successful treatments. Brain-specific glycans and glycopeptides are therefore interesting candidates to include in such screens.

We are now closer to achieving a functional understanding of protein glycosylation with quantitative in-depth site-specific analysis of the glycoproteome from the brain and CSF. These data need to be processed so that correlations may be revealed that link glycopeptides, glycoproteins, glycosites, glyco-networks and AD phenotypes.

## Figures and Tables

**Table 1:** Summary of N-glycan structures determined from N-glycomics studies to have significantly altered levels in CSF from AD patients compared to controls. Only N-glycans with fully determined structures are shown. The N-glycans are drawn as cartoons according to CFG nomenclature. (F), female; (M), male; **↑**, increased in AD; **↓**, decreased in AD; **↑→** increased in a subgroup of the AD patients; **↓→**, decreased in a subgroup of the AD patients.

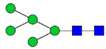	↑	Schedin-Weiss [71]	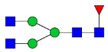	↑(F)	Gizaw [69]
	↑→	Palmigiano [68]			
	↑(F); ↑(M)	Gizaw [69]			
	↓(F); ↓(M)	Cho [70]			
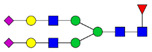	↓→	Schedin-Weiss [71]	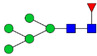	↓(F); ↓(M)	Cho [70]
	↓→	Palmigiano [68]			
	↑(F); ↑(M)	Gizaw [69]			
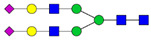	↑→	Schedin-Weiss [71]	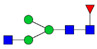	↑(F)	Cho [70]
	↑(F); ↑(M)	Gizaw [69]			
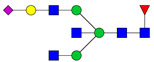	↑	Schedin-Weiss [71]	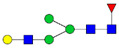	↓(M)	Cho [70]
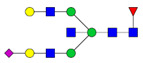	↑	Schedin-Weiss [71]	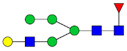	↓(M)	Cho [70]
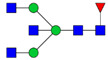	↑	Schedin-Weiss [71]	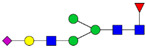	↓(M)	Cho [70]
	↑→	Palmigiano [68]			
	↑(F); ↑(M)	Gizaw [69]			
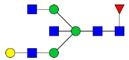	↑	Schedin-Weiss [71]	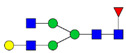	↓(F)	Cho [70]
	↑→	Palmigiano [68]			
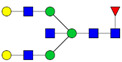	↑	Schedin-Weiss [71]	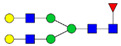	↓(F)	Cho [70]
	↑(F)	Cho [70]			
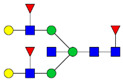	↑→	Schedin-Weiss [71]	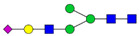	↓(F)	Cho [70]
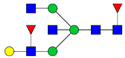	↑	Schedin-Weiss [71]	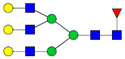	↓(F)	Cho [70]
	↑→	Palmigiano [68]			
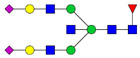	↑(F)	Gizaw [69]	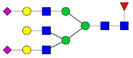	↑(M)	Cho [70]
	↓(F)	Cho [70]			
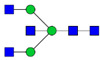	↑→	Palmigiano [68]	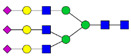	↓(F)	Cho [70]
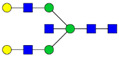	↓(F); ↓(M)	Cho [70]	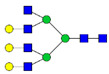	↑(F)	Cho [70]
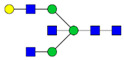	↑(F)	Cho [70]	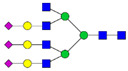	↓(F)	Cho [70]
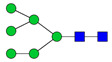	↑(F); ↑(M)	Gizaw [69]			

**Table 2:** Summary of fragmentation types and instruments commonly used for analysis of glycopeptides. CID, collision-induced dissociation; EThcD, electron transfer/higher energy dissociation; FT-ICR, Fourier transform ion cyclotron resonance; HCD, higher-energy C trap dissociation, sceHCD; stepped collision energy HCD.

Fragmentation Type	Instrument	Polarity	Glycopeptide Information	Glycomics Information	Suitable for Glycoproteomics	Suitable for Glycomics	References
Resonance activation CID	Ion trap (linear, 3D)	+/−	Glycan identification but only blind mass of peptide	Glycan composition, sequence and structure are possible with MSn. Mostly glycosidic bond cleavage	not optimal	yes	[81]
Beam type CID/HCD	Q-TOF, Orbitraps,	+/−	Normal Q-TOF CID and HCD yield peptide identification but only composition and partial sequence of glycans and usually not the glycosite. Energy resolved Q-TOF MS2 can yield peptide identification, site identification and glycan composition sequence/structure.	Glycan composition, sequence and structure is possible at MS2 or MS3 due to higher energy than CID.	Yes, if using energy-resolved MS2 in Q-TOF and sceHCD (see below)	yes	[82,83,84]
Energy-resolved HCD/stepped collision energy HCD (sceHCD)	Orbitrap	+/−	Peptide identification, site identification, glycan composition and sequence/structure.	Glycan composition, sequence and structure are possible.	yes	yes	[85,86]
EThcD	Orbitrap Fusion/Lumos	+/−	Peptide identification, site identification, glycan composition and sequence/structure.	Complementary fragmentation with ETD and HCD is usually needed to yield more glycan fragments.	yes	Complementary fragmentation with ETD and HCD is usually not needed to yield more glycan fragments	[87,88]
Conventional ETD/ECD	Iontrap, Orbitrap, FT-ICR	+/−	Peptide identification, site identification, blind mass of glycan	Mostly cross ring cleavage	Yes, but better if combined with CID or HCD (see below)	Yes, but better if combined with CID or HCD (see below)	[89,90,91,92,93]
Conventional ETD/ECD combined with CID or HCD	Iontrap, FT-ICR	+/−	Peptide identification, site identification, glycan composition and sequence/structure	Glycan composition, sequence and structure is possible	Yes	Yes	[94,95]

**Table 3:** A selection of glycomics and glycoproteomics software tools mentioned in this review.

Search Engine	Academic or Commercial	Special MS Data Requirements	Comment	Reference
Protein prospector	Academic			[101]
MSFragger-Glyco	Academic		Open search, Spectral library reduces the search space	[102]
PGlyco 2.0 and 3.0	Academic	Suitable for sceHCD		[86,103]
Byonic	Commercial		Glycan modification search and wildcard (open) search	[104]
GlycReSoft	Academic			[105]
GlycoPAT	Academic			[106]

## Data Availability

Not applicable.

## Supplementary Materials

The following are available online at https://www.mdpi.com/article/10.3390/biom11060858/s1, Table S1: Summary of CSF N-glycomics and N-glycoproteomics studies referred to in this review. The main findings are listed together with methodological details such as biological sample, LC-MS approach, glycoproteome coverage and glycomic techniques.
